# Interaction Between AtCML9 and AtMLO10 Regulates Pollen Tube Development and Seed Setting

**DOI:** 10.3389/fpls.2020.01119

**Published:** 2020-07-23

**Authors:** Qian Zhang, Congcong Hou, Yudan Tian, Mitianguo Tang, Changxin Feng, Zhijie Ren, Jiali Song, Xiaohan Wang, Tiange Li, Mengou Li, Wang Tian, Jinlong Qiu, Liangyu Liu, Legong Li

**Affiliations:** ^1^ College of Life Sciences, Capital Normal University, and Beijing Key Laboratory of Plant Gene Resources and Biotechnology for Carbon Reduction and Environmental Improvement, Beijing, China; ^2^ State Key Laboratory of Plant Genomics, Institute of Microbiology, Chinese Academy of Sciences, Beijing, China

**Keywords:** CML9, MLO10, pollination, pollen tube, stigma, recognition

## Abstract

In higher-plant reproduction, the compatibility of pollen tube germination in the pistil is essential for successful double fertilization. It has been reported that *Mildew Locus O* (*MLO*) family gene *NTA* (*MLO7*), expressing in synergid cells, can correctly guide pollen tubes. However, the molecular mechanism underlying the interacting partners to MLOs in the fertilization is still unknown. In our study, we identified the direct protein interaction between CML9 and MLO10 within a non-canonical CaMBD. In GUS reporter assays, *CML9* expresses in a high level in pollens, whereas *MLO10* can be specifically detected in stigma which reaches up to a peaking level before fertilization. Therefore, the spatio-temporal expression patterns of *MLO10* and *CML9* are required for the time-window of pollination. When we observed the pollen germination *in vitro*, two *cml9* mutant alleles dramatically reduced germination rate by 15% compared to wild-type. Consistently, the elongation rate of pollen tubes *in planta* was obviously slow while manually pollinating *cml9-1* pollens to *mlo10-1* stigmas. Additionally, *cml9-1 mlo10-1* double mutant alleles had relatively lower rate of seed setting. Taken together, protein interaction between MLO10 and CML9 is supposed to affect pollen tube elongation and further affect seed development.

## Introduction

Reproductive process is critical for flowering plants. After physically attaching to the stigma epidermal, pollen grains successively adhere, hydrate, and germinate. Then pollen tubes penetrating along the style are guided to the ovary to release the sperms (Zheng et al., 2018). Those steps require the matured male and female organs to communicate and recognize each other, and any molecule inhibiting male–female recognition leads to incompatibility. During pollination, pollen–stigma recognition usually depends on specific protein–protein interactions, which is classified into several types of compatibilities (Knox et al., 1976; Dumas and Knox, 1983). For example, when expressing the *SRK* (*S-*locus receptor kinase) and *SCR* (*S-*locus cysteine rich protein) genes in the stigma and pollen respectively, SCR–SRK protein interaction leads to unsuccessful germination of stigma-attached pollens (Schopfer et al., 1999; Takasaki et al., 2000; Nasrallah et al., 2002). In the GSI (Gametophytic Self-incompatibility) type, while S-RNase in the stigma recognizes and interacts with SFB (*S*-locus F-Box) from the germinated pollen, it results in defective of pollen tube elongation (McClure and Franklin-Tong, 2006; Hiscock and Allen, 2008). In pollination stages, specific protein interactions determine the compatibility or incompatibility of pollens with stigma and other pistil parts.

When a pollen tube is guided to the ovule, several recognition combinations of ligand-receptors like LURE1-MIK/MDIS1, RALF34-BUPS-ANX have been characterized (Wang et al., 2016; Ge et al., 2017). In *Mildew Locus* *O* (*MLO*) gene family, recent studies have also identified a fertilization related gene *MLO7* (also named *NOTIA*) localized in synergid cells. In *mlo7* mutant, the pollen tube cannot be correctly guided to the embryo sac, which further fails to burst the tip and release sperm cells to continue the double fertilization (Kessler et al., 2010; Jones and Kessler, 2017). Based on phylogenetic analysis, *MLO10*, expressed in flower organs, is considered to be a *MLO7*-related gene. However, it remains unclear whether MLO10 is involved in fertilization (Wuest et al., 2010; Jones et al., 2017). Therefore, we aim to investigate MLO10 as well as its interaction partners that may mediate recognition between the pollen tube and stigma or embryo sac through direct protein–protein interaction.

Based on bioinformatic analysis, it has been found that one of the domains called CaMBD (Calmodulin binding domain) is significantly enriched in the C-terminal of 15 MLO proteins (Knox et al., 1976; Feechan et al., 2008; Appiano et al., 2015). These indicate that MLOs may interact with calmodulins to regulate the reproduction process. Accumulated pieces evidence proves that calcium plays a critical role in plant fertilization (Iwano et al., 2009; Qin and Yang, 2011; Hepler et al., 2012; Wudick et al., 2018; Zhang et al., 2020). Calmodulins (CaMs) or Calmodulins like (CMLs) proteins have been identified to regulate plant development and stress mediated reactions (Cheval et al., 2013; Zhu et al., 2015). Several CaMs and CMLs have been found to be involved in pollen germination and tube elongation. For example, CaM2 forms a complex with CNGC8/18 to control pollen tube elongation through modulating calcium oscillations at the tips (Pan et al., 2019). Meanwhile, mutation of *CaM2* significantly inhibits pollen germination (Landoni et al., 2010; Leba et al., 2012). CML25 was also reported to affect pollen tube development and seed setting (Wang et al., 2015).

In conclusion, although some components of MLOs or CaM/CMLs have been reported to be involved in plant reproduction, the direct link between these two-family proteins is missing. In our study, we have identified the new protein interacting complex MLO10–CML9 which is involved in pollen germination, pollen tube elongation as well as seed setting. We also investigate and speculate how the direct interaction between stigma-expressed MLO10 and pollen-expressed CML9 regulates reproductive physiology.

## Materials and Methods

### Plant Materials and Growth Conditions

Experiments with *Arabidopsis thaliana* were performed on ecotype Columbia-0. The knock-out mutant lines *cml9-1* (SALK_126787C) and *cml9-2* (SALK_006380C), were obtained from the ABRC (https://www.arabidopsis.org/abrc/) as described (Heyer et al., 2018). Seeds were stratified for 2 d at 4°C in darkness and then grown under a 16-h-light/8-h-dark cycle at a photon fluence rate of approximately 120 μmol·m^−2^·s^−1^ during the day. Plants were kept at 22°C with approximately 60% humidity. The plates containing 0.5 strength Murashige and Skoog media and 1% (w/v) sucrose solidified with 0.6% (w/v) Agar. After 7 days, the seedlings were transplanted into the soil under the same light cycle, temperature, and humidity.

### CRISPR-Cas9 Mediated Gene Editing Events of *MLO10* in *Arabidopsis*

The method for *MLO10* editing was performed as Gao et al. (2016) described. *PHEN401* is used as *MLO10* gene-editing vector. The CRISPR/Cas9 constructs were transformed into *Arabidopsis* wild-type Columbia-0 through floral dipping. T1 plants were selected on 25 µg·L^−1^ hygromycin B. Genomic DNA samples extracted from leaf tissues of 2-week-old T1 plants were used as templates for PCR. The PCR product amplified with targeting site-specific primers was digested using restriction enzyme XhoI. Putative mutations should produce XhoI-resistant band. Then results were verified by sequencing the PCR products. Cas9-free T3 seeds were isolated. Our experiments were performed on the Cas9-free lines *mlo10-1* and *mlo10-2* (**Supplementary Figure 3A**). The primers used in these cloning procedures are listed in **Table 1**.

**Table 1:** Primer sequences used in the experiment.

Primer	Sequence
AtMLO10::3242-F	CGGGATCCTTAGTCAATATCATTAGCAGGA
AtMLO10::3242-R	GGAATTCTATGGCCACAAGATGCTTTTGG
AtCML9::3228-F	GGGGTACCATGGCGGATGCTTTCACAGAT
AtCML9::3228-R	CGGGATCCCTAATAAGAGGCAGCAATCATC
AtMLO10::pBI101-F	GCGTCGACGCTGATTGCTATGCCAAGTCCAC
AtMLO10::pBI101-R	CGGGATCCGGTAGCCAAAAGAAATC
AtCML9::PBI101-F	CGGGATCCAACAAAATTGGAGCGTTTGAG
AtCML9::PBI101-R	CCCCCCGGGATCTTCGATCACAAAGAAAAG
AtMLO10::1302-F	GAAGATCTGATGGCCACAAGATGCTTTTGG
AtMLO10::1302-R	GAAGATCTACGTCAATATCATTAGCAGGA
AtCML9::1302-F	GAAGATCTGATGGCGGATGCTTTCACAGAT
AtCML9::1302-R	GACTAGTATAAGAGGCAGCAATCATCAT
AtMLO10-P1-F	CGGAATTCCAGATGGGTTCAAACATGAAG
AtMLO10-P1-R	CGGGATCCTTAGTCAATATCATTAGCAGG
AtMLO10-P2-F	CGGAATTCCAGATGGGTTCAAACATGAAG
AtMLO10-P2-R	CGGGATCCGAAAGAGCGAAACGGAAAGAA
AtMLO10-P3-F	CGGAATTCGCAAAAGCGTTGAAGAAATGG
AtMLO10-P3-R	CGGGATCCTTAGTCAATATCATTAGCAGG
AtMLO10-P4-F	CGGAATTCGCAAAAGCGTTGAAGAAATGG
AtMLO10-P4-R	CGGGATCCGAAAGAGCGAAACGGAAAGAA
AtMLO10-P5-F	CGGAATTCAGGGTTGGTGATCAGAACACA
AtMLO10-P5-R	CGGGATCCTTAGTCAATATCATTAGCAGG
Salk_006380C-F	TTGTAGGAATCAGCATCGGAT
Salk_006380C-R	ACTGATTTTTGGTTCTTCAGA
Salk_126787C-F	TGAGCGATGTTGACATCTTTG
Salk_126787C-R	ACAATACTTTTGGTTTGGTT
AtMLO10-F	ATGGCCACAAGATGCTTTTGGT
AtMLO10-R	TTAGTCAATATCATTAGCAGGA
AtCML9-F	ATGGCGGATGCTTTCACAGAT
AtCML9-R	CTAATAAGAGGCAGCAATCAT
Actin2-F	CACTGTGCCAATCTACGAGGGT
Actin2-R	ACAAACGAGGGCTGGAACAAG
AtMLO10::pHEN401-F	ATTGTGTCTCCGTCCTCCTCGAGA
AtMLO10::pHEN401-R	AAACTCTCGAGGAGGACGGAGACA
Crispr-AtMLO10-F	AAACGGACTTTCAGTCAACAC
Crispr-AtMLO10-R	TTGAAAACTCGTAATCATGAG

### Yeast Two-Hybrid Assays

Coding regions of *MLO10-CT*-(P1-P5) and *CaM2*, *CaM4*, *CaM6*, *CaM7*, *CML8*, *CML9*, *CML10* sequences were cloned into the *pCBKT7* vector and *pGADT7* vector respectively. Primers are listed in **Table 1**. Then the plasmids (*MLO10-CT::BD* and *CaMs/CMLs::AD*, *MLO10-CT::BD* fragments P1–P5 and *CML9::AD*, respectively) were co-transformed into the yeast AH109 strain, using the method of PEG/LiAc. Transformants were selected on the SD medium lacking Leu and Trp. After 3 days of growth, the haploid cells were transferred to a selected medium containing 1.5 mM 3-AT but lacking Leu, Trp, His. The X-Gal filter assay was done as described earlier and modified (Moeini-Naghani and Navaratnam, 2016). The primers used in these cloning procedures are listed in **Table 1**.

### Subcellular Localization and BiFC Assay

We used *Arabidopsis* mesophyll protoplasts for the GFP and BiFC transient expression assay. To generate the *35S*::*MLO10*-*GFP*, *35S*::*CML9*-*GFP* (Green Fluorescent Protein) plasmid, CDS sequences (without stop codon) were inserted into the *pCAMBIA1302* vector. For BiFC vectors, sequences of *MLO10* and *CML9* were inserted into *pSAT1-nVenus-C* (*pE3242*) and *pSAT1-cCFP-C* (*pE3228*) respectively. Primers are listed in **Table 1**. Plasmids were transferred into *Arabidopsis thaliana* mesophyll protoplasts as described (Yoo et al., 2007). After 16 h of incubation at 23°C in the dark, the GFP or CFP, YFP signals were detected using a confocal laser scanning microscopy (LSM 780; Carl Zeiss).

### Construction of Transgenic Lines and Analyses of GUS Activity

About 5-week-old Col wild type plants with several mature flowers were transformed with the *MLO10pro::GUS* and C*ML9pro::GUS* constructs in *PBI101* respectively, *via* the *Agrobacterium tumefaciens* strain GV3101 by using the floral dip method (Bent and Clough, 1998). The primers used in these cloning procedures are listed in **Table 1**. T1 transgenic plant seedlings were selected on 1/2 MS plates containing 50 μg/L kanamycin. Examination of GUS activity in transgenic seedlings was performed as described (Rosado et al., 2012). Samples were fixed for 20 min in ice-cold 90% (vol/vol) acetone, were washed three times (5 min per wash) with ice-cold phosphate buffer [100 mM sodium phosphate (pH 7), 10 μM EDTA, 0.1% Triton X-100, 2 mM K_4_Fe(CN)_6._3H_2_O, 2 mM K_3_Fe(CN)_6_], and were stained in X-gluc (5-bromo-4-chloro-3-indolyl-*β*-D-glucuronic acid) solution (2 mM X-gluc in the same phosphate buffer) at 37°C in darkness, check the GUS activity every 1 h. After the assay these tissues were washed in 70% ethanol, 80% ethanol, 90% ethanol, and finally in 100% ethanol. The plant tissues after GUS staining were photographed with microscope (Discovery.V20, ZEISS).

### Aniline Blue Staining Assay

Aniline blue staining of pollen tubes in pistils was performed as described (Jiang et al., 2005) and modified. The pre-emasculated mature wild-type and *mlo10-1* stigma were pollinated either with wild-type or *cml9-1* pollen respectively. The pollinated pistils were collected 4 hap (hour after pollination) and briefly fixed in a fixing solution of ethanol:acetic acid (3:1) for 2 h at room temperature. The fixed pistils were washed three times with distilled water and treated in softening solution of 8 M NaOH overnight. Then, the pistil tissues were washed in distilled water and stained in aniline blue solution (0.1% aniline blue in 0.1 M K_4_Fe(CN)_6_•3H_2_O buffer, pH 11) for 3 to 5 h in the dark. The stained pistils were observed and photographed with a Leica DMRA fluorescence microscope. The images were digitized, and the pollen tube lengths were measured using ImageJ 1.52a software.

### Analysis of Pollen Germination and Pollen Tubes Length

*In vitro* germination assays were modified as described (Fan et al., 2001; Mouline et al., 2002). Pollens were collected by tapping the open anthers on solid agar and then incubated in the dark at 28°C. The solid pollen germination medium consisted of 10 μg ·ml^−1^ inositol, 1.5 mM H_3_BO_3_, 5 mM MES, 10 mM CaCl_2_, 1 mM KCl, 0.8 mM MgSO_4_, 1% agar, and 20% sucrose, pH 5.8. For analysis of pollen germination and pollen tube growth *in vitro*, samples were incubated for 6 h. Pollen tubes were photographed with microscope (Discovery.V20, ZEISS). The images were digitized, and the pollen tube lengths were measured using ImageJ 1.52a software.

## Results

### Physical Interaction Between CML9 and MLO10

**Figure 1:**
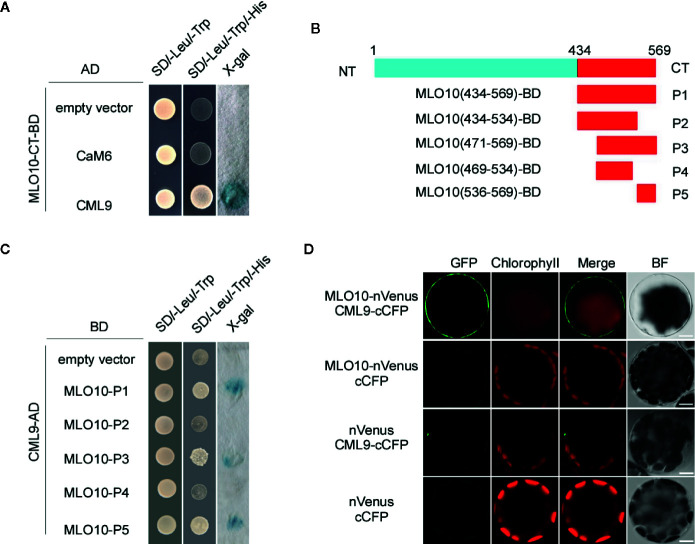
Protein Interaction of CML9 and MLO10. **(A)** Yeast two-hybrid assay of calmodulins (CaMs)/CaM-like proteins (CMLs) and the C-terminal of MLO10 (MLO10-CT). The combination of pGBKT7-MLO10-CT and empty pGADT7 vectors was used as a negative control. SD/-Trp/-Leu represents synthetic dextrose minimal medium without tryptophan and leucine; SD/-Trp/-Leu/-His indicates synthetic dextrose minimal medium without tryptophan, leucine, and histidine. Growth of yeast on SD/-Trp/-Leu/-His plate indicates interaction between the two tested proteins. **(B)** Diagram representing various domains of the MLO10-CT (P1–P5), which were cloned and used as prey during yeast two-hybrid screening. **(C)** Yeast two-hybrid analysis of CaM1 with various fragments of MLO10-CT. **(D)** Bimolecular fluorescence complementation (BiFC) analysis between MLO10 and CML9 in *Arabidopsis* mesophyll protoplasts. Vectors encoding MLO10-nVenus, CML9-cCFP, and nVenus were co-expressed in various combinations in *Arabidopsis* protoplasts. Green fluorescence (GFP), red fluorescence (Chlorophyll), merged fluorescence of green and red (Merge), and bright field (BF) were detected, respectively. Scale bars, 10 μm.

MLO family proteins contain one CaM-binding domain (CaMBD) in most higher plants. It has been reported that HvMLO1 can bind to CaMs *via* CaMBDs at the C-terminal (CT) cytosolic regions. We focused on the possible interacting partners especially the calcium sensor proteins to MLO10 in *Arabidopsis* *thaliana*. However, CaM binding has not been studied well with MLO10, and the specific binding sites between CaMs and MLO10 remain unknown. First, based on the bioinformatic prediction by TMHMM 2.0, MLO10-CT is supposed to localize at the cytoplasm. Thus, taking MLO10-CT as bait, we conducted a yeast two hybrid (Y2H) screen with *Arabidopsis* CaMs and CMLs. Interestingly, we identified the interaction event between MLO10 and CML9. When co-expressing MLO10-CT-BD and CML9-AD, the yeast colony became blue by adding X-gal into the selection media (**Figure 1A**), which indeed confirms the direction interaction of MLO10-CML9. However, no obvious colony was observed when combining MLO10 and CML9 isoforms like CaM2, CaM4, CaM6, CaM7 and CML8, CML10 (**Figure 1A**, **Supplementary Figure 1**), which suggests the MLO10–CML9 interaction could be specific. Next, we wanted to map the specific interaction domain of MLO10. When aligning amino acids of several MLOs, generally the divergent domain ranges from 430 to 590 aa (MLO C-terminal), and the putative CaMBD, which was marked with arrows (**Supplementary Figures 2A, C**), may be involved in determining the specificity of protein interactions. Therefore, we divided MLO10-CT which has 136 aa (434–569 aa) into five truncation fragments P1–5 (**Figure 1B**). Surprisingly, through the X-gal based Y2H assay, the results showed that P1, P3, and P5 can interact with CML9 (**Figure 1C**), which suggests the shared P5 fragment (536–569 aa) is the critical domain of the C-terminal of MLO10. We concluded that the P5 domain on the C-terminal of MLO10, but not the canonical CaMBD, is responsible for specific interaction with CML9.

We further confirmed MLO10–CML9 interaction in BiFC (Bimolecular Fluorescence Complementation) assay. When co-expressing MLO10-nVenus and CML9-cCFP, green fluorescence signal was detected significantly in the plasma membrane of *Arabidopsis* protoplasts (**Figure 1D**), which was obviously different compared to the controls like nVenus + cCFP, nVenus + CML9-cCFP and MLO10-nVenus + cCFP. It indicates that MLO10 recruits and physically interacts with CML9 in the plasma membrane (**Figure 1D**). Taken together, we have identified the direct interaction of MLO10–CML9 in both Y2H and BiFC systems.

### Subcellular Localization of CML9 and MLO10

**Figure 2:**
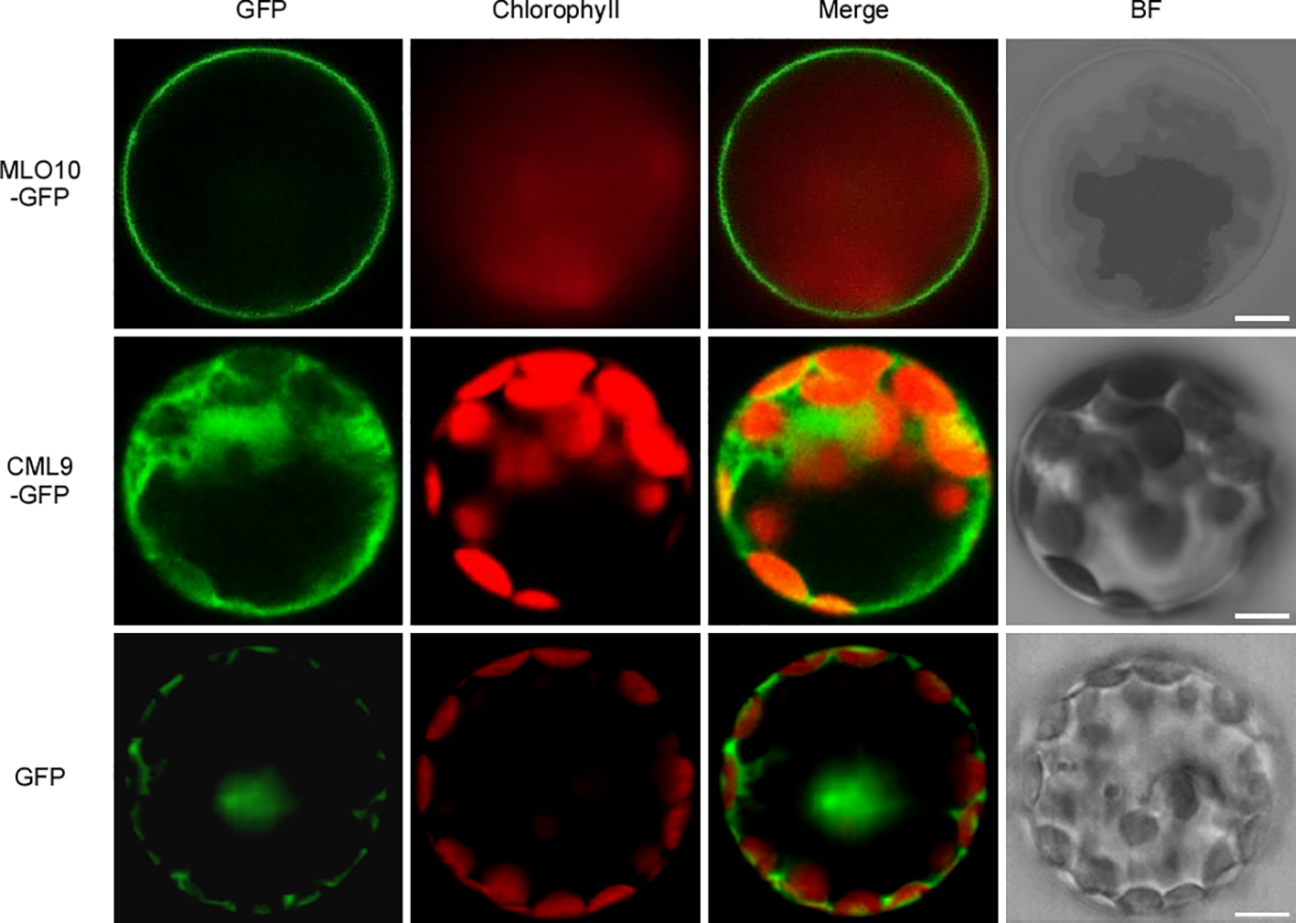
Subcellular localization of MLO10 and CML9 in *Arabidopsis* mesophyll protoplast. AtMLO10-GFP and AtCML9-GFP proteins were transiently expressed in protoplasts under the control of the cauliflower mosaic virus 35S promoter. Green fluorescence (GFP), red fluorescence (Chlorophyll), yellow fluorescence (merged fluorescence of green and red), and bright field (BF) were detected, respectively. Scale bars, 10 μm.

To investigate the subcellular localization of CML9 and MLO10, we generated *35S:MLO10-GFP* and *35S:CML9-GFP* vectors which were individually introduced into *Arabidopsis* protoplasts. Only the GFP protein as control is localized in the nucleus, cytoplasm, and membrane (**Figure 2**). We can observe the obvious signal of the fusion protein MLO10-GFP mainly in the cell membrane and very weak signal in some other membrane-attached parts (**Figure 2**). However, the CML9-GFP expressed alone is mainly localized in the cytoplasm (**Figure 2**). Therefore, combined with the observation of BiFC results above, we concluded that the protein interaction between membrane-localized MLO10 and cytoplasm-localized CML9 occurs in the plant cell (**Figure 1D**).

### *MLO10* and *CML9* Are Co-Expressed in Flowers Within a Spatio-Temporal Specific Manner

**Figure 3:**
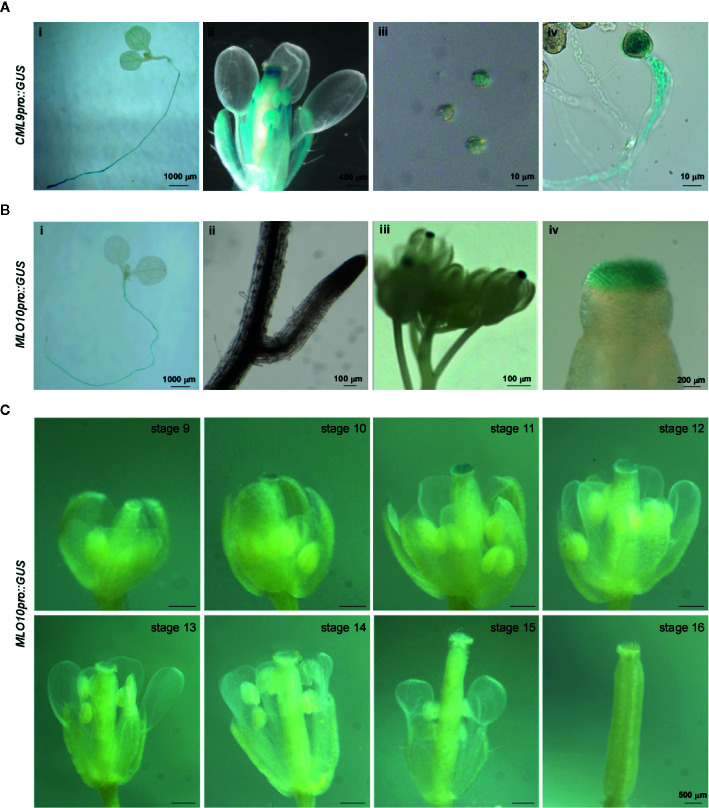
Tissue specific expression pattern of MLO10 and CML9. **(A)** GUS staining of *CML9pro::GUS* transgenic *Arabidopsis* lines in whole seedling (i), lateral root (ii), inflorescence (iii). and stigma (iv). **(B)** GUS staining of *MLO10pro::GU*S transgenic *Arabidopsis* lines in whole seedling (i), inflorescence (ii), pollen (iii), and pollen tube (iv). **(C)** Expression pattern of *MLO10pro::GUS* in flower development stages 9–16. Scale bars, 500 μm.

In order to test the tissue specific expression pattern of *CML9* and *MLO10*, we fused *CML9* and *MLO10* promoters with *GUS* reporter gene respectively. The results showed that *CML9* is generally expressed in the root tips and leaf vasculature bundle, especially in the filament and anther of flowers (**Figure 3A**). The reporter GUS signal driven by *CML9* promoter was stained not only in matured pollens released from the opening anther, but also highly in the germinated pollens and pollen tubes (**Figures 3A-ii, -iv**). This observation demonstrated that *CML9* probably plays an important role in the pollen and pollen tube development during pollination.

According to the GUS staining assay, *MLO10* staining signals were restricted to the root, leaf vasculature bundle, and stigma (**Figure 3B**). When we further analyzed GUS signal in different developmental stages of flowers, the results suggest that *MLO10* expression gets to a peaking level at stage 11 and displays relatively low expression levels in other flower developmental stages (**Figure 3C**). Flower developmental stages 13–14 are considered as the time-window of pollination recognition between matured pollen and stigma papilla, which is in line with the equal height of stamen and carpel [**Figure 1C** and (Alvarez-Buylla et al., 2010)]. In addition, *MLO10* expression was also observed in siliques previously (Chen et al., 2006).

Taken together, the specific spatio-temporal expression pattern led us to hypothesize that *MLO10*, expressing slightly early before pollinaton, may get ready to interact with later expressed *CML9*.

### Mutation of *CML9* Reduced the Rates of Pollen Germination and Pollen Tube Elongation

**Figure 4:**
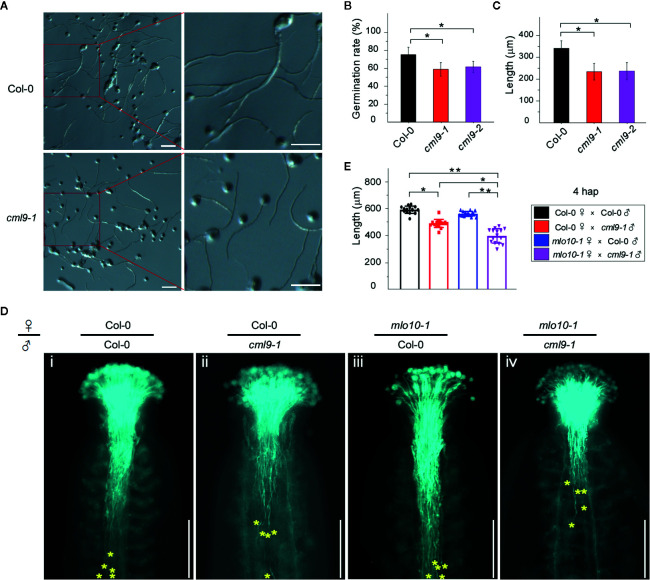
Mutation of *CML9* affect pollen germination and pollen tube elongation. **(A)** *In vitro* test of pollen germination in wild-type and *cml9-1* (4hap). Scale bars, 100 μm. **(B)** Quantification of pollen germination rate of different genotypes from **(A)**. Data are shown as the mean ± s.e.m. *n* = 20. Two-tailed Students’ *t*-test. **(C)** Quantification of pollen tube length of Col-0, *cml9-1*, and *cml9-2*. Data are shown as the mean ± s.e.m. *n* = 100. Two-tailed Students’ *t*-test. **(D)** Manual pollination assay showed different pollen tube lengths in Col-0 ♀ × Col-0 ♂, Col-0 ♀ × *cml9-1* ♂, *mlo10-1* ♀ × Col-0 ♂, *mlo10-1* ♀ × *cml9-1* ♂ (4 hap). Yellow stars show top 5 of the furthest pollen tubes. Scale bars, 250 μm. **(E)**. Statistics of D. Data are shown as the mean ± s.e.m. *n* = 15. Two-tailed Students’ *t*-test. *p < 0.05, **p < 0.01.

Next, we investigated the physiological function of the MLO10–CML9 protein-protein interaction on affecting reproductive development. Because *CML9* was highly expressed in the pollen and pollen tube (**Figure 1B**), we firstly measured the germination rates of two *cml9* mutant alleles and Col-0 pollens. The results showed that after 4 h *in vitro* germination assay the rate of Col-0 was about 75%, but *cml9-1* and *cml9-2* mutants significantly reduced to 60% (**Figures 4A, B**). Consistently, at 4 hap (hour after pollination), the pollen tube lengths in *cml9-1* and *cml9-2* were severely shorter than that in Col-0 (**Figure 4C**). Collectively, the mutation of *CML9* affects the germination rate of pollens and elongation rate of pollen tubes. To further test whether it is also true *in planta*, we carried out the manual pollination through four combinations that take *cml9-1* and Col-0 as male-parent, *mlo10-1* and Col-0 as female-parent. In line with the observation *in vitro* germination, the pollen tube penetration rate of Col-0 ♀ × *cml9-1* ♂ was obliviously reduced by about 14% as compared with Col-0 ♀ × Col-0 ♂ (**Figures 4D, E**). Interestingly, *mlo10-1* ♀ × *cml9-1* ♂ showed lowest pollen tube penetration rate among the four tested combinations (**Figures 4D, E**), suggesting that both CML9 and MLO10 contribute to pollen tube elongation *in planta*, which is possibly mediated through MLO10–CML9 interacting module.

### Genetic Interaction of *MLO10* and *CML9* Affects Seed Setting Rate

**Figure 5:**
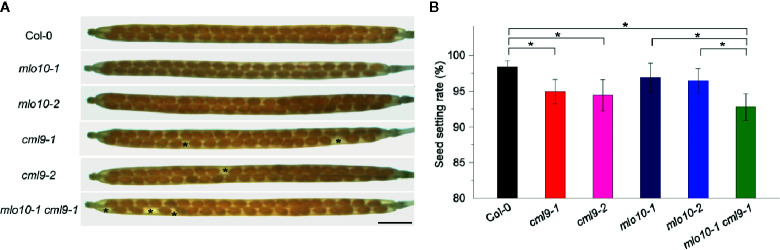
*cml9-1 mlo10-1* double mutant reduced seed setting. **(A)** Seed setting rates in Col-0, *cml9-1*, *cml9-2*, *mlo10-1, mlo10-2*, and *cml9-1 mlo10-1*. Scale bars, 1,500 μm. **(B)** Statistics of **(A)**. Data are shown as the mean ± s.e.m. *n* = 40. Two-tailed Students’ *t*-test. p < 0.05.

We further asked whether the pollen tube elongation mediated by MLO10–CML9 may affect later developmental stages like silique development in *Arabidopsis*. The self-pollination siliques were checked among the materials of Col-0, *cml9-1*, *cml9-2*, *mlo10-1*, *mlo10-2*, and *cml9-1 mlo10-1*. Phenotypically, a few seeds were obviously defective in *cml9-1*, *cml9-2*, and *cml9-1 mlo10-1* (**Figure 5A**). We found that seed setting rates in *cml9-1*, *cml9-2*, and *cml9-1 mlo10-1* were less by a reduction rate around 5% compared to Col-0 (**Figures 5A, B**). Taken together, MLO10–CML9 module could affect the developmental process of seed setting.

## Discussion

In this study, we identified a protein complex MLO10–CML9 which may be involved in pollen–stigma recognition and affect pollen tube elongation and seed setting. These two proteins display the maternal- and paternal-specific expression manner during pollination period. Their genetic interaction further demonstrates the function of MLO10–CML9 in plant reproduction physiology.

### MLOs May Have General Function for Recognition

MLO family members have divergent biological functions which mediate many communications, such as the touching effect between roots and environmental materials (Chen et al., 2009; Bidzinski et al., 2014), pathogen attack to leaves (Consonni et al., 2006; Acevedo-Garcia et al., 2017), recognition between pollen tube and synergid cells (Kessler et al., 2010; Jones and Kessler, 2017), as well as pollen–stigma communication in this study. In *brief*, we may consider MLOs as plant endogenous monitors to various environmental or internal stimuli. Therefore, mutation of MLOs may block or inhibit the recognition process in plant.

In Barley, mutation of *HvMLOs* inhibits the pathogen attack of powdery mildew by inducing local leaf neurosis (Consonni et al., 2006). In *Arabidopsis mlo7* mutant, pollen tubes cannot be correctly guided to the embryo sac (Kessler et al., 2010), indicating that this recognition may be mediated by the interaction of MLO7 and some unknown protein. Interestingly, in this study, we identified a new interacting module MLO10–CML9 which could be related to pollen–stigma recognition. Especially, genetic mutations of *MLO10* in stigma as well as *CML9* in pollen result in significant reduction of pollen germination and pollen tube elongation rates, suggesting MLO10–CML9 contributes to fertilization. Our future work will figure out the detailed molecular interactions of MLO10 and CML9 *in planta*, as well as the possibility of redundancy modules to MLO10-CML9.

Taken together, MLO members interacting with their partners may function in sensing environmental stimuli and cell–cell communications. The previous and our studies will provide a new insight to deeply understand the functions of MLO family.

### Interaction of Calmodulins and MLOs Provide a New Module in Regulating Reproductive Physiology and Stress Response

Calcium oscillations at pollen tube tips are important for maintaining its polarity growth (Iwano et al., 2009; Qin and Yang, 2011; Hepler et al., 2012; Wudick et al., 2018). CNGC8/18 as calcium channels interact with CaM2 to form a complex to encode calcium oscillations at pollen tube tips (Pan et al., 2019). Mutation of *CaM2* leads to low rate of pollen germination (Landoni et al., 2010), and *cml25* mutant was also reported to reduce rates of pollen germination *in vitro* and *in planta* (Wang et al., 2015). Those observations are compatible with our results in *cml9-1* and *cml9-2* mutants. Therefore, we conclude that CaM/CMLs may have an important and basic role in pollen–stigma recognition as well as polarity growth of pollen tubes. MLO10–CML9 complex may further create a new connection among the membrane protein, calcium sensor, and calcium oscillations.

Considering the stress responses, CML37 positively promotes ABA synthesis in response to drought stress (Wang et al., 2015), whereas CML42 negatively regulates plant defense by downregulation of JA response genes (Vadassery et al., 2012). CML9 acts as a positive player in response to bacterial effector triggered immunity (ETI) (Leba et al., 2012). A recent study identified that a protein complex CaM–CNGC2–CNGC4 mediated the early event of calcium-dependent ETI process (Tian et al., 2019).

In barley, calmodulin can interact with the C-terminal domain of MLO protein to regulate pathogen defense (Kim et al., 2002b). Especially, extracellar calmodulin can bind to a plant cell membrane (Wang et al., 2009), suggesting that camodulin could function as a mobile signal across cells. In our study, we found CML9 interacts with MLO10 which is involved in fertilization and seed setting. Therefore, in future work it will be worth to investigate the direct connection between MLO10–CML9 complex mediated calcium and ROS signaling (Zhang et al., 2020), which can further lead to deeply understanding the physiology of double fertilization.

## Data Availability Statement

The original contributions presented in the study are included in the article/supplementary material; further inquiries can be directed to the corresponding authors.

## Author Contributions

LeL, QZ and LiL conceived and designed the project. QZ and CH performed most of the research and data analysis, and YT, MT, ZR, CF, JS, XW, TL, ML and WT prepared the genetic materials and observed the phenotype and collected data. LeL and LiL drafted the manuscript and LeL, LiL, and JQ revised the manuscript.

## Conflict of Interest

The authors declare that the research was conducted in the absence of any commercial or financial relationships that could be construed as a potential conflict of interest.

## References

[B1] Acevedo-Garcia J. Gruner K. Reinstadler A. Kemen A. Kemen E. Cao L. (2017). The powdery mildew-resistant *Arabidopsis mlo2 mlo6 mlo12* triple mutant displays altered infection phenotypes with diverse types of phytopathogens. Sci. Rep. 7 (1), 9319. 10.1038/s41598-017-07188-7 28839137 PMC5570895

[B2] Alvarez-Buylla E. R. Benitez M. Corvera-Poire A. Chaos Cador A. de Folter S. Gamboa de Buen A. (2010). Flower development. Arabidopsis Book 8, e0127. 10.1199/tab.0127 22303253 PMC3244948

[B3] Appiano M. Catalano D. Santillan Martinez M. Lotti C. Zheng Z. Visser R. G. (2015). Monocot and dicot MLO powdery mildew susceptibility factors are functionally conserved in spite of the evolution of class-specific molecular features. BMC Plant Biol. 15, 257. 10.1186/s12870-015-0639-6 26499889 PMC4620714

[B4] Bent A. F. Clough S. J. (1998). “Agrobacterium Germ-Line Transformation: Transformation of Arabidopsis without Tissue Culture,” in Plant Molecular Biology Manual. Eds. Gelvin S. B. Schilperoort R. A. (Dordrecht: Springer Netherlands), 17–30.

[B5] Bhat R. A. Miklis M. Schmelzer E. Schulze-Lefert P. Panstruga R. (2005). Recruitment and interaction dynamics of plant penetration resistance components in a plasma membrane microdomain. Proc. Natl. Acad. Sci. U.S.A. 102 (8), 3135–3140. 10.1073/pnas.0500012102 15703292 PMC549507

[B6] Bhat R. A. Lahaye T. Panstruga R. (2006). The visible touch: in planta visualization of protein-protein interactions by fluorophore-based methods. Plant Methods 2:12. 10.1186/1746-4811-2-12 16800872 PMC1523328

[B7] Bidzinski P. Noir S. Shahi S. Reinstadler A. Gratkowska D. M. Panstruga R. (2014). Physiological characterization and genetic modifiers of aberrant root thigmomorphogenesis in mutants of *Arabidopsis thaliana MILDEW LOCUS O* genes. Plant Cell Environ. 37 (12), 2738–2753. 10.1111/pce.12353 24738718

[B8] Chen Z. Hartmann H. A. Wu M. J. Friedman E. J. Chen J. G. Pulley M. (2006). Expression analysis of the *AtMLO* gene family encoding plant-specific seven-transmembrane domain proteins. Plant Mol. Biol. 60 (4), 583–597. 10.1007/s11103-005-5082-x 16525893

[B9] Chen Z. Noir S. Kwaaitaal M. Hartmann H. A. Wu M. J. Mudgil Y. (2009). Two seven-transmembrane domain *MILDEW RESISTANCE LOCUS O* proteins cofunction in *Arabidopsis* root thigmomorphogenesis. Plant Cell 21 (7), 1972–1991. 10.1105/tpc.108.062653 19602625 PMC2729597

[B10] Cheval C. Aldon D. Galaud J. P. Ranty B. (2013). Calcium/calmodulin-mediated regulation of plant immunity. Biochim. Biophys. Acta 1833 (7), 1766–1771. 10.1016/j.bbamcr.2013.01.031 23380707

[B11] Consonni C. Humphry M. E. Hartmann H. A. Livaja M. Durner J. Westphal L. (2006). Conserved requirement for a plant host cell protein in powdery mildew pathogenesis. Nat. Genet. 38 (6), 716–720. 10.1038/ng1806 16732289

[B12] Dumas C. Knox R. B. (1983). Callose and determination of pistil viability and incompatibility. Theor. Appl. Genet. 67 (1), 1–10. 10.1007/BF00303914 24258474

[B13] Elliott C. Muller J. Miklis M. Bhat R. A. Schulze-Lefert P. Panstruga R. (2005). Conserved extracellular cysteine residues and cytoplasmic loop-loop interplay are required for functionality of the heptahelical MLO protein. Biochem. J. 385 (Pt 1), 243–254. 10.1042/BJ20040993 15352871 PMC1134693

[B14] Fan L. M. Wang Y. F. Wang H. Wu W. H. (2001). In vitro *Arabidopsis* pollen germination and characterization of the inward potassium currents in *Arabidopsis* pollen grain protoplasts. J. Exp. Bot. 52 (361), 1603–1614. 10.1093/jexbot/52.361.1603 11479325

[B15] Feechan A. Jermakow A. M. Torregrosa L. Panstruga R. Dry I. B. (2008). Identification of grapevine *MLO* gene candidates involved in susceptibility to powdery mildew. Funct. Plant Biol. 35 (12), 1255–1266. 10.1071/FP08173 32688872

[B16] Gao X. Chen J. Dai X. Zhang D. Zhao Y. (2016). An Effective Strategy for Reliably Isolating Heritable and *Cas9*-Free *Arabidopsis* Mutants Generated by CRISPR/Cas9-Mediated Genome Editing. Plant Physiol. 171 (3), 1794–1800. 10.1104/pp.16.00663 27208253 PMC4936589

[B17] Ge Z. Bergonci T. Zhao Y. Zou Y. Du S. Liu M. C. (2017). *Arabidopsis* pollen tube integrity and sperm release are regulated by RALF-mediated signaling. Science 358 (6370), 1596–1600. 10.1126/science.aao3642 29242234 PMC5964610

[B18] Hepler P. K. Kunkel J. G. Rounds C. M. Winship L. J. (2012). Calcium entry into pollen tubes. Trends Plant Sci. 17 (1), 32–38. 10.1016/j.tplants.2011.10.007 22104406

[B19] Heyer M. Scholz S. S. Voigt D. Reichelt M. Aldon D. Oelmuller R. (2018). Herbivory-responsive calmodulin-like protein CML9 does not guide jasmonate-mediated defenses in *Arabidopsis thaliana* . PloS One 13 (5), e0197633. 10.1371/journal.pone.0197633 29768484 PMC5955546

[B20] Hiscock S. J. Allen A. M. (2008). Diverse cell signalling pathways regulate pollen-stigma interactions: the search for consensus. New Phytol. 179 (2), 286–317. 10.1111/j.1469-8137.2008.02457.x 19086285

[B21] Iwano M. Entani T. Shiba H. Kakita M. Nagai T. Mizuno H. (2009). Fine-tuning of the cytoplasmic Ca^2+^ concentration is essential for pollen tube growth. Plant Physiol. 150 (3), 1322–1334. 10.1104/pp.109.139329 19474213 PMC2705041

[B22] Jiang L. Yang S. L. Xie L. F. Puah C. S. Zhang X. Q. Yang W. C. (2005). *VANGUARD1* encodes a pectin methylesterase that enhances pollen tube growth in the *Arabidopsis* style and transmitting tract. Plant Cell 17 (2), 584–596. 10.1105/tpc.104.027631 15659637 PMC548828

[B23] Jones D. S. Kessler S. A. (2017). Cell type-dependent localization of MLO proteins. Plant Signal Behav. 12 (11), e1393135. 10.1080/15592324.2017.1393135 29039994 PMC5703261

[B24] Jones D. S. Yuan J. Smith B. E. Willoughby A. C. Kumimoto E. L. Kessler S. A. (2017). *MILDEW RESISTANCE LOCUS O* Function in Pollen Tube Reception Is Linked to Its Oligomerization and Subcellular Distribution. Plant Physiol. 175 (1), 172–185. 10.1104/pp.17.00523 28724621 PMC5580752

[B25] Kessler S. A. Shimosato-Asano H. Keinath N. F. Wuest S. E. Ingram G. Panstruga R. (2010). Conserved molecular components for pollen tube reception and fungal invasion. Science 330 (6006), 968–971. 10.1126/science.1195211 21071669

[B26] Kim M. C. Lee S. H. Kim J. K. Chun H. J. Choi M. S. Chung W. S. (2002a). Mlo, a modulator of plant defense and cell death, is a novel calmodulin-binding protein. Isolation and characterization of a rice Mlo homologue. J. Biol. Chem. 277 (22), 19304–19314. 10.1074/jbc.M108478200 11904292

[B27] Kim M. C. Panstruga R. Elliott C. Muller J. Devoto A. Yoon H. W. (2002b). Calmodulin interacts with MLO protein to regulate defence against mildew in barley. Nature 416 (6879), 447–451. 10.1038/416447a 11919636

[B28] Knox R. B. Clarke A. Harrison S. Smith P. Marchalonis J. J. (1976). Cell recognition in plants: Determinants of the stigma surface and their pollen interactions. Proc. Natl. Acad. Sci. U.S.A. 73 (8), 2788–2792. 10.1073/pnas.73.8.2788 16592342 PMC430745

[B29] Landoni M. De Francesco A. Galbiati M. Tonelli C. (2010). A loss-of-function mutation in *Calmodulin2* gene affects pollen germination in *Arabidopsis thaliana* . Plant Mol. Biol. 74 (3), 235–247. 10.1007/s11103-010-9669-5 20683641

[B30] Leba L. J. Cheval C. Ortiz-Martin I. Ranty B. Beuzon C. R. Galaud J. P. (2012). CML9, an *Arabidopsis* calmodulin-like protein, contributes to plant innate immunity through a flagellin-dependent signalling pathway. Plant J. 71 (6), 976–989. 10.1111/j.1365-313X.2012.05045.x 22563930

[B31] McClure B. A. Franklin-Tong V. (2006). Gametophytic self-incompatibility: understanding the cellular mechanisms involved in “self” pollen tube inhibition. Planta 224 (2), 233–245. 10.1007/s00425-006-0284-2 16794841

[B32] Moeini-Naghani I. Navaratnam D. S. (2016). Yeast Two-Hybrid Screening to Test for Protein-Protein Interactions in the Auditory System. Methods Mol. Biol. 1427, 95–107. 10.1007/978-1-4939-3615-1_6 27259923

[B33] Mouline K. Very A. A. Gaymard F. Boucherez J. Pilot G. Devic M. (2002). Pollen tube development and competitive ability are impaired by disruption of a Shaker K^+^ channel in *Arabidopsis* . Genes Dev. 16 (3), 339–350. 10.1101/gad.213902 11825875 PMC155331

[B34] Nasrallah M. E. Liu P. Nasrallah J. B. (2002). Generation of self-incompatible *Arabidopsis thaliana* by transfer of two *S* locus genes from *A. lyrata* . Science 297 (5579), 247–249. 10.1126/science.1072205 12114625

[B35] Pan Y. Chai X. Gao Q. Zhou L. Zhang S. Li L. (2019). Dynamic Interactions of Plant CNGC Subunits and Calmodulins Drive Oscillatory Ca^2+^ Channel Activities. Dev. Cell 48 (5), 710–725 e715. . 10.1016/j.devcel.2018.12.025 30713075

[B36] Qin Y. Yang Z. (2011). Rapid tip growth: insights from pollen tubes. Semin. Cell Dev. Biol. 22 (8), 816–824. 10.1016/j.semcdb.2011.06.004 21729760 PMC3210868

[B37] Rosado A. Li R. van de Ven W. Hsu E. Raikhel N. V. (2012). *Arabidopsis* ribosomal proteins control developmental programs through translational regulation of auxin response factors. Proc. Natl. Acad. Sci. U.S.A. 109 (48), 19537–19544. 10.1073/pnas.1214774109 23144218 PMC3511758

[B38] Schopfer C. R. Nasrallah M. E. Nasrallah J. B. (1999). The male determinant of self-incompatibility in *Brassica* . Science 286 (5445), 1697–1700. 10.1126/science.286.5445.1697 10576728

[B39] Takasaki T. Hatakeyama K. Suzuki G. Watanabe M. Isogai A. Hinata K. (2000). The *S* receptor kinase determines self-incompatibility in *Brassica* stigma. Nature 403 (6772), 913–916. 10.1038/35002628 10706292

[B40] Tian W. Hou C. Ren Z. Wang C. Zhao F. Dahlbeck D. (2019). A calmodulin-gated calcium channel links pathogen patterns to plant immunity. Nature 572 (7767), 131–135. 10.1038/s41586-019-1413-y 31316205

[B41] Vadassery J. Reichelt M. Hause B. Gershenzon J. Boland W. Mithofer A. (2012). CML42-mediated calcium signaling coordinates responses to Spodoptera herbivory and abiotic stresses in *Arabidopsis* . Plant Physiol. 159 (3), 1159–1175. 10.1104/pp.112.198150 22570470 PMC3387702

[B42] Wang Q. Chen B. Liu P. Zheng M. Wang Y. Cui S. (2009). Calmodulin binds to extracellular sites on the plasma membrane of plant cells and elicits a rise in intracellular calcium concentration. J. Biol. Chem. 284 (18), 12000–12007. 10.1074/jbc.M808028200 19254956 PMC2673269

[B43] Wang S. S. Diao W. Z. Yang X. Qiao Z. Wang M. Acharya B. R. (2015). *Arabidopsis thaliana* CML25 mediates the Ca^2+^ regulation of K^+^ transmembrane trafficking during pollen germination and tube elongation. Plant Cell Environ. 38 (11), 2372–2386. 10.1111/pce.12559 25923414

[B44] Wang T. Liang L. Xue Y. Jia P. F. Chen W. Zhang M. X. (2016). A receptor heteromer mediates the male perception of female attractants in plants. Nature 531 (7593), 241–244. 10.1038/nature16975 26863186

[B45] Wudick M. M. Portes M. T. Michard E. Rosas-Santiago P. Lizzio M. A. Nunes C. O. (2018). CORNICHON sorting and regulation of GLR channels underlie pollen tube Ca^2+^ homeostasis. Science 360 (6388), 533–536. 10.1126/science.aar6464 29724955

[B46] Wuest S. E. Vijverberg K. Schmidt A. Weiss M. Gheyselinck J. Lohr M. (2010). *Arabidopsis* female gametophyte gene expression map reveals similarities between plant and animal gametes. Curr. Biol. 20 (6), 506–512. 10.1016/j.cub.2010.01.051 20226671

[B47] Yoo S. D. Cho Y. H. Sheen J. (2007). *Arabidopsis* mesophyll protoplasts: a versatile cell system for transient gene expression analysis. Nat. Protoc. 2 (7), 1565–1572. 10.1038/nprot.2007.199 17585298

[B48] Zhang M. J. Zhang X. S. Gao X. Q. (2020). ROS in the Male-Female Interactions During Pollination: Function and Regulation. Front. Plant Sci. 11:177. 10.3389/fpls.2020.00177 32180782 PMC7059789

[B49] Zheng Y. Y. Lin X. J. Liang H. M. Wang F. F. Chen L. Y. (2018). The Long Journey of Pollen Tube in the Pistil. Int. J. Mol. Sci. 19 (11), 3529 10.3390/ijms19113529 PMC6275014 30423936

[B50] Zhu X. Dunand C. Snedden W. Galaud J. P. (2015). CaM and CML emergence in the green lineage. Trends Plant Sci. 20 (8), 483–489. 10.1016/j.tplants.2015.05.010 26115779

