# Supplementary material for: Interaction Between AtCML9 and AtMLO10 Regulates Pollen Tube Development and Seed Setting
Source: Front Plant Sci. 2020 Jul 23;11:1119. doi: 10.3389/fpls.2020.01119 (PMC7394235; doi:10.3389/fpls.2020.01119)
Supplement: Supplementary file 1 [file DataSheet_1.docx]

**Supplementary Figure 1**


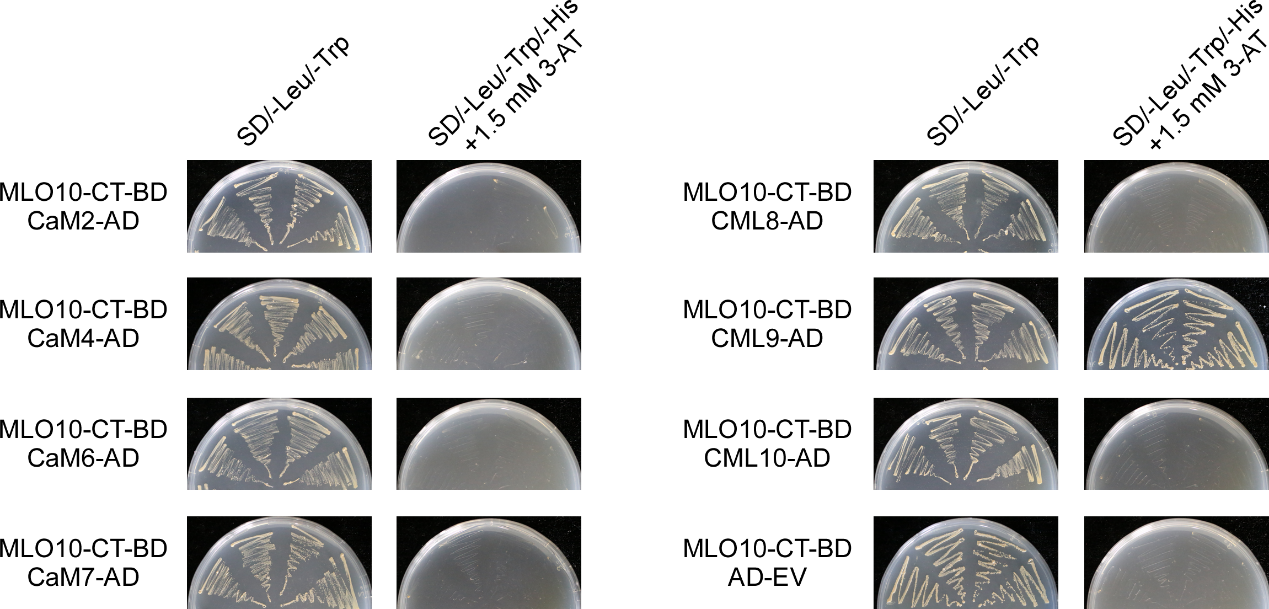


SUPPLEMENTARY FIGURE 1 | Interaction between MLO10 and CaMs/CMLs. Co-expression MLO10-CT (Bait) and CaM2, CaM4, CaM6, CaM7, CML8, CML9, CML10, AD-Vector (Prey) in to AH109 strain, respectively. The transformants were cultured on SD-Leu/-Trp medium, and then transferred to SD-Leu/-Trp/-His (1.5 mM 3-AT) medium. Growth of yeast on SD/-Trp/-Leu/-His medium indicates interaction between the two tested proteins.

**Supplementary Figure 2**

**
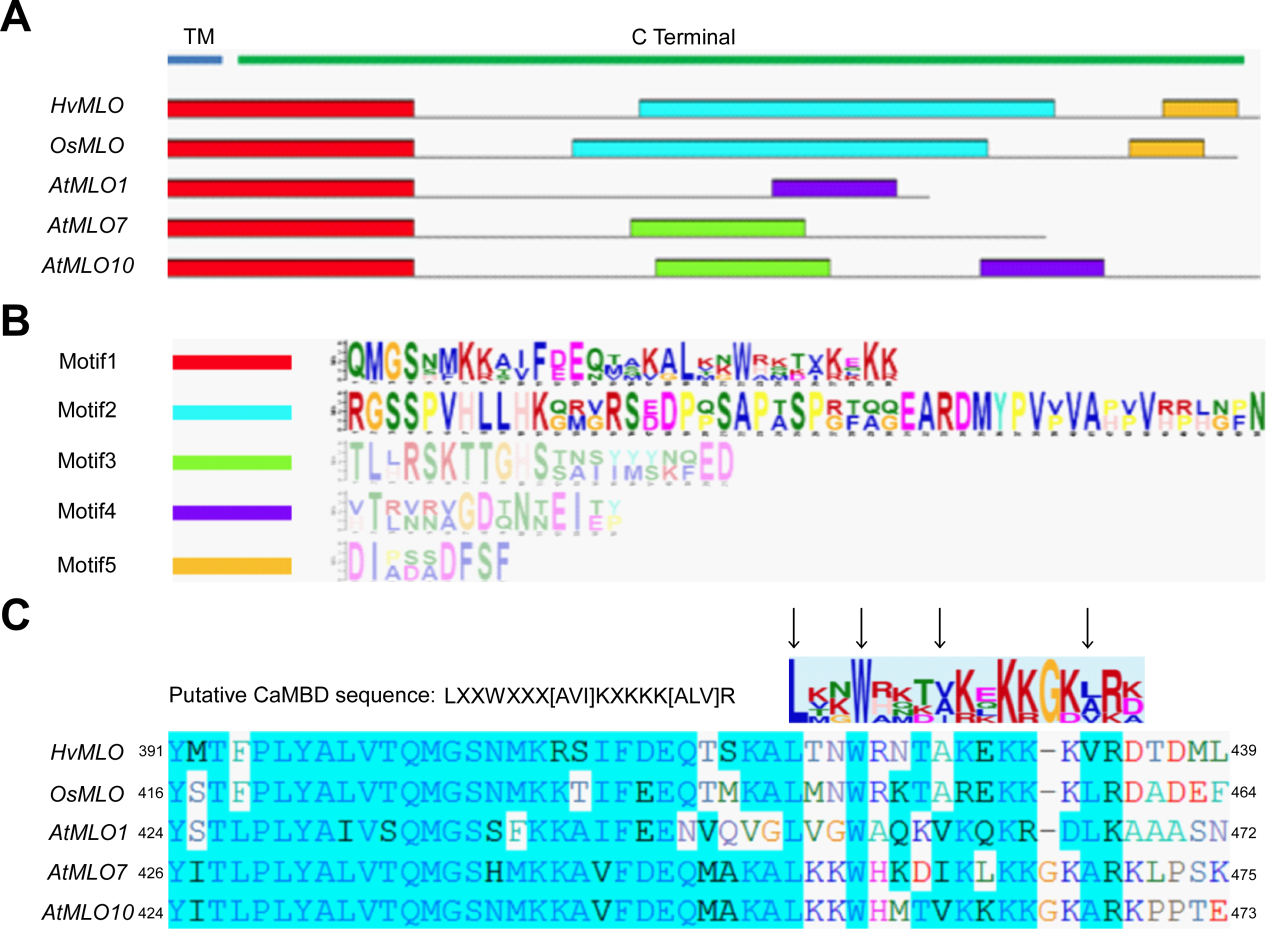
**

SUPPLEMENTARY FIGURE 2 | Analysis of the CaM-binding domain sequences of MLO10 protein. (A, B) Motif analysis of plant MLO families. The amino acid sequences of MLO proteins were obtained from GenBank^TM^ data base searches. Motif Locations (A) and Discovered Motifs (B) were aligned by the MEME software (*http://meme-suite.org*). The GenBankTM accession numbers used in building the phylogenetic tree are as follows: HvMLO (Z83834), OsMLO (AF388195), AtMLO1 (Z95352), and AtMLO7 (AF369568), AtMLO10 (AF369571). Hv, *Hordeum vulgare;* Os, *Oryza sativa*; At, *Arabidopsis thaliana*. C. Alignment of the putative CaMBD sequence of MLO10 (Leu453 to Ala467) with the corresponding regions of MLO families that were demonstrated interacting with CaMs. HvMLO (Kim et al., 2002b; Bhat et al., 2005; Elliott et al., 2005; Bhat et al., 2006); OsMLO (Kim et al., 2002a); AtMLO1 (Kim et al., 2002b). The putative Ca^2+^-dependent CaM-binding motif of hydrophobic amino acids were indicated with black arrows (1-4-8-14 motif, as Kim described (Kim et al., 2002a)).

**Supplementary Figure 3**


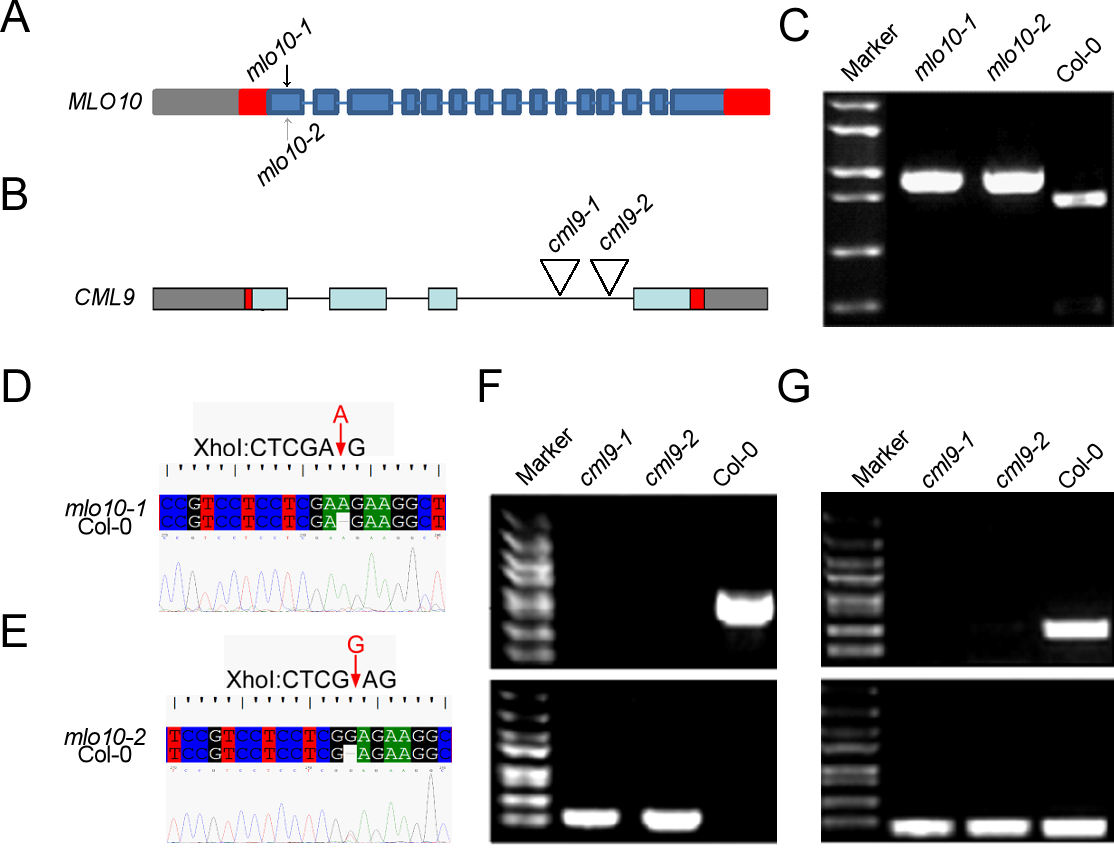


SUPPLEMENTARY FIGURE 3 | Mutation screening of *MLO10* and *CML9*. (A) Structure of *MLO10* gene. The black and gray arrows showed the CRISPR-Cas9 mediated gene-editing positions of *mlo10-1* and *mlo10-2*, respectively. (B) Structure of *CML9* gene. T-DNA Insertion of *cml9-1* (SALK_126787C) and *cml9-2* (SALK_006380C). (C) *MLO10* genomic DNA from *mlo10-1* and *mlo10-2* could not be digested by restriction enzyme XhoI. (D, E) Mutations of *MLO10* in two different alleles (*mlo10-1* and *mlo10-2*). (F) Upper figure shows genomic DNA band of *CML9*, lower figure shows insertion of T-DNA. (G) Upper figure shows *CML9* band at RNA level, lower figure shows transcription level of *ACTIN2*.
